# Propionate Increases Hepatic Pyruvate Cycling and Anaplerosis and Alters Mitochondrial Metabolism*

**DOI:** 10.1074/jbc.M116.720631

**Published:** 2016-03-21

**Authors:** Rachel J. Perry, Candace B. Borders, Gary W. Cline, Xian-Man Zhang, Tiago C. Alves, Kitt Falk Petersen, Douglas L. Rothman, Richard G. Kibbey, Gerald I. Shulman

**Affiliations:** From the Departments of ‡ Internal Medicine,; ** Cellular and Molecular Physiology, and; ¶ Radiology and Biomedical Imaging, and; the ‡‡ Howard Hughes Medical Institute, Yale University School of Medicine, New Haven, Connecticut 06519,; the ‖ Department of Biomedical Engineering, Yale University, New Haven, Connecticut 06519, and; § The Novo Nordisk Foundation Center for Basic Metabolic Research, University of Copenhagen, Copenhagen DK 1017, Denmark

**Keywords:** glucagon, gluconeogenesis, isotopic tracer, liver metabolism, pyruvate kinase, epinephrine

## Abstract

In mammals, pyruvate kinase (PK) plays a key role in regulating the balance between glycolysis and gluconeogenesis; however, *in vivo* regulation of PK flux by gluconeogenic hormones and substrates is poorly understood. To this end, we developed a novel NMR-liquid chromatography/tandem-mass spectrometry (LC-MS/MS) method to directly assess pyruvate cycling relative to mitochondrial pyruvate metabolism (*V*_Pyr-Cyc_/*V*_Mito_) *in vivo* using [3-^13^C]lactate as a tracer. Using this approach, *V*_Pyr-Cyc_/*V*_Mito_ was only 6% in overnight fasted rats. In contrast, when propionate was infused simultaneously at doses previously used as a tracer, it increased *V*_Pyr-Cyc_/*V*_Mito_ by 20–30-fold, increased hepatic TCA metabolite concentrations 2–3-fold, and increased endogenous glucose production rates by 20–100%. The physiologic stimuli, glucagon and epinephrine, both increased hepatic glucose production, but only glucagon suppressed *V*_Pyr-Cyc_/*V*_Mito_. These data show that under fasting conditions, when hepatic gluconeogenesis is stimulated, pyruvate recycling is relatively low in liver compared with *V*_Mito_ flux and that liver metabolism, in particular pyruvate cycling, is sensitive to propionate making it an unsuitable tracer to assess hepatic glycolytic, gluconeogenic, and mitochondrial metabolism *in vivo*.

## Introduction

Pyruvate kinase (PK)^3^ catalyzes the irreversible final step of the glycolytic pathway in mammals, the dephosphorylation of phosphoenolpyruvate (PEP) to pyruvate. Because of the large free energy drop of PEP hydrolysis by PK, two enzymatic reactions (PC and PEPCK, each consuming an ATP equivalent) are required to make PEP from pyruvate in gluconeogenic tissues. Consequently, PK, PC, and PEPCK reactions are highly regulated steps essential for gluconeogenesis, and under fasting conditions, when hepatic gluconeogenesis is contributing to the entirety of endogenous glucose production (EGP), the liver would be expected to limit futile cycling through pyruvate kinase.

In this regard, it was surprising that several studies have reported extremely high rates of hepatic pyruvate cycling, which were 2–4 times the rate of citrate synthase flux (*V*_CS_) in both rodents and humans, when using [1,2,3-^13^C_3_]propionate as a metabolic tracer (1–5). Such high rates of pyruvate cycling would use a significant fraction of the ATP available to the hepatocyte, placing it in a metabolically precarious position. We hypothesized that propionate itself, when used at the concentrations necessary to interpret *in vitro* ^13^C isotope labeling, may actually promote increased hepatic pyruvate cycling and explain the relatively high rates of hepatic pyruvate cycling observed under these conditions.

**FIGURE 1.**
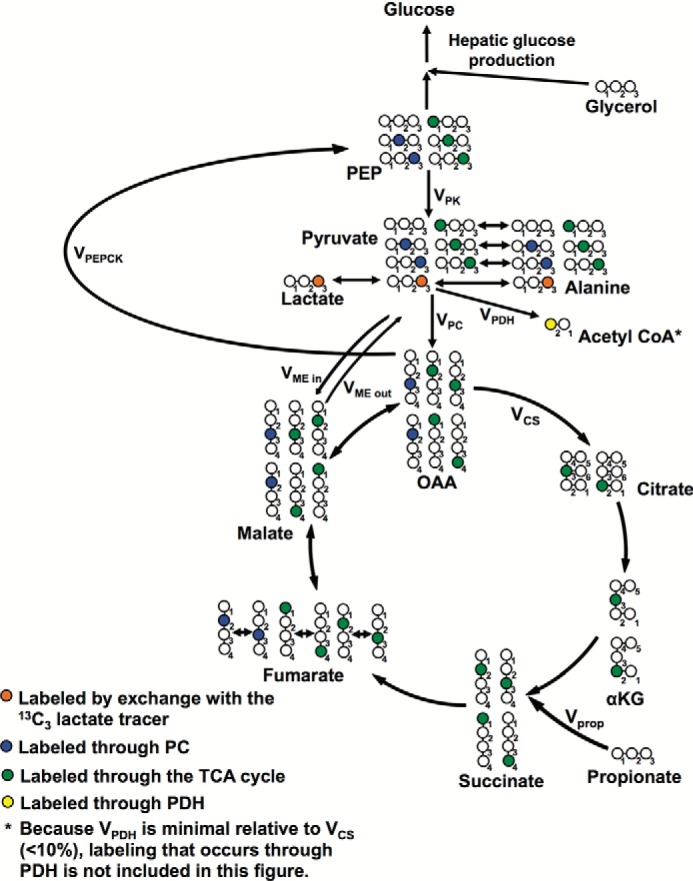
**Flux modeling scheme using [3-^13^C]lactate as a tracer.**

To assess this possibility, we developed a novel combined NMR-LC-MS/MS method to measure *V*_PK + ME_/*V*_PC + PDH_, *i.e.* hepatic pyruvate cycling (*V*_PK_ + *V*_ME_) relative to mitochondrial pyruvate metabolism (*V*_PC_ + *V*_PDH_) in awake rats (where *V*_PK_ refers to PK flux; *V*_ME_ refers to malic enzyme flux in the direction of pyruvate synthesis; *V*_PC_ refers to anaplerosis via pyruvate carboxylase; and *V*_PDH_ refers to pyruvate oxidation via pyruvate dehydrogenase), and for simplicity we refer to this as *V*_Pyr-Cyc_/*V*_Mito_. This ratio was measured from ^13^C labeling patterns that develop in liver when using [3-^13^C]lactate as a tracer (Fig. 1) (6, 7) and is calculated using the ratio of [2-^13^C]alanine/[2-^13^C]malate.

Using this combined NMR-LC-MS/MS approach, we found that *V*_Pyr-Cyc_ was only 6% of *V*_Mito_ in overnight fasted rats and that glucagon, but not epinephrine, suppressed *V*_Pyr-Cyc_/*V*_Mito_ despite similar increases in hormone concentrations and EGP rates. Furthermore, we also found that propionate infusion dose-dependently increased *V*_Pyr-Cyc_/*V*_Mito_, gluconeogenesis and anaplerotic metabolites by 2–3-fold in liver. In principle, metabolic tracers should not alter the processes that they are attempting to measure; thus, these data demonstrate on multiple levels the unsuitability of [1,2,3-^13^C_3_]propionate as a metabolic tracer for hepatic metabolism.

## Experimental Procedures

### 

#### 

##### Animals

Sprague-Dawley rats (∼350 g) were purchased from Charles River Laboratories and fed normal chow (Harlan 2018). Rats underwent surgery under general isoflurane anesthesia to place polyethylene catheters in the carotid artery, jugular vein, and/or antrum of the stomach (PE50, PE90, and PE50 tubing, respectively, Instech Solomon). After 1 week of recovery, rats were fasted overnight and underwent infusion studies as described below. All animal protocols were approved by the Yale University Animal Care and Use Committee.

##### Tracer Studies

Overnight fasted rats received a primed-continuous 120-min co-infusion of [3-^13^C]lactate (prime 120 μmol/(kg-min) for 5 min and 40 μmol/(kg-min) for the remaining 115 min) and [3-^3^H]glucose (0.45 μCi/(kg-min) for 5 min and 0.15 μCi/(kg-min) for the remaining 115 min) through their arterial catheter. After 120 min of infusion, a blood sample was drawn from the venous catheter and immediately transferred to a heparin-coated tube (Beckman Coulter), and the rats were sacrificed by intravenous pentobarbital. Their livers were freeze-clamped *in situ* in tongs pre-cooled in liquid N_2_ and stored at −80 °C pending further analysis.

Rats undergoing intra-arterial propionate infusion studies were infused with sodium propionate (low dose = 333 μmol/kg = 2.8 μmol/(kg-min); high dose = 667 μmol/kg = 5.6 μmol/(kg-min)) for 120 min concurrently with an infusion of [3-^13^C]lactate and [3-^3^H]glucose at the rates listed above. The doses of propionate were selected to administer less than (2–4, 8) or similar to (5) the total amount of sodium propionate used in previous studies, where it was given as an oral or intraperitoneal (i.p.) bolus. Blood and livers were isolated after 120 min as described above, and hepatic fluxes were calculated as described below.

In the intragastric propionate treatment studies, propionate (200 μmol/kg in water) was administered through a catheter surgically placed 7 days previously in the antrum of the stomach. Blood samples were taken through an intravenous catheter every 15 min following propionate administration. A subset of rats were euthanized at the time of peak plasma propionate concentration (30 min), and blood was taken from the portal vein for measurement of propionate concentrations. Separate groups of rats were infused with glucagon (5 ng/(kg-min)) or epinephrine (2 μg/(kg-min)) for 120 min concurrently with an infusion of [3-^13^C]lactate and [3-^3^H]glucose at the rates listed above.

Malic enzyme activity was inhibited by an intraperitoneal injection of hydroxymalonate, a small molecule ME inhibitor (200 mg/kg, Sigma, a dose selected to achieve concentrations higher than the effective dose to suppress ME flux *in vivo* in isolated rat heart mitochondria (9)), in groups of control, glucagon-treated, epinephrine-treated, and high dose propionate-treated rats. Immediately after the injection, a 120-min infusion of [3-^13^C]lactate and [3-^3^H]glucose was initiated, and rats were sacrificed 2 h after treatment with ME inhibitor.

In the hyperinsulinemic-euglycemic clamp studies, rats received a primed continuous infusion of insulin (200 milliunits/kg prime at time 0, 4 milliunits/ (kg-min) continuous) and [3-^3^H]glucose (0.15 μCi/(kg-min)), as well as variable 20% dextrose to maintain euglycemia (5.8–6.5 mm) through their arterial catheter. Blood samples (∼50 μl whole blood) were taken through their venous catheter every 15 min, and the glucose infusion rate was adjusted to maintain euglycemia (100–110 mg/dl).

##### LC-MS/MS Analysis

Hepatic propionyl-CoA content was measured by LC-MS/MS. ∼100 mg of liver tissue was disrupted using a TissueLyser (Qiagen) in 1 ml of ice-cold 10% trichloroacetic acid. An internal standard (5 nmol of [1,2-^13^C_2_]acetyl-CoA) was added before homogenization. The samples were mixed on a rotating shaker for 30 min at 4 °C and centrifuged at 4000 × *g* for 5 min at 4 °C. The supernatant was then loaded on a C18 cartridge, which had been preconditioned by 3 ml of methanol and 4 ml of 1 mm HCl, and then washed with 3 ml of 1 mm HCl and 1 ml of distilled water. The flow-through was discarded at this point. Finally, the CoAs were eluted from the cartridge with 2 ml of ethanol/water (65–35%, v/v, containing 0.1 m ammonium acetate) and 2 ml of 50% HPLC-grade methanol in water. The samples were dried in a SpeedVac for the minimum time necessary (∼6 h), resuspended in 100 μl of distilled water, and transferred to LC-MS/MS vials. The AbSCIEX 6500 QTRAP was used for LC-MS/MS analysis using an electrospray ionization source with positive-ion detection. Propionyl-CoA and [1,2-^13^C_2_]acetyl-CoA were selectively monitored in multiple reaction monitoring mode, with ion pairs 824.0/317.0 and 812.0/305.1, respectively. Concentrations were measured by comparing the ratio of [1,2-^13^C_2_]acetyl-CoA to propionyl-CoA peak areas against those of a standard curve.

To measure aspartate, malate, and succinate concentrations and enrichments by LC/MS/MS, ∼100 mg of tissue were weighed out, and a solution containing 0.1 μmol of the related internal standards ([1,2,3,4-^13^C_4_]succinic acid, [1,2,3,4-^13^C_4_]malic acid, and [1,2,3,4-^13^C_4_]aspartic acid) was added. We then homogenized the liver tissue in 500 μl of methanol using a TissueLyser. After centrifuging and filtering through a NANOSEP filter tube to remove debris, we used LC-MS/MS (AbSCIEX 6500 QTRAP with a Shimadzu ultrafast liquid chromatography system in negative-ion MRM mode) to monitor the following ion pairs: succinate and [1,2,3,4-^13^C_4_]succinate, 117/99 and 121/103, respectively; malate and [1,2,3,4-^13^C_4_]malate, 133/115 and 137/119, respectively; and aspartate and [1,2,3,4-^13^C_4_]aspartate, 132/115 and 136/119, respectively.

To measure total alanine enrichment, a 50-μl aliquot of the NMR extract was dried in the SpeedVac (Savant), and 75 μl of *n*-butanol 4 n HCl was added. The samples were then heated for 30 min at 60 °C and dried overnight in a 60 °C vacuum oven. The following day, the samples were reacted with 100 μl of trifluoroacetic acid in methylene chloride (1:7 v/v), and alanine enrichment was determined by GC/MS (chemical ionization mode, *m*/*z* 242 and 243), with the average *m*/*z* ratio of three liver samples from rats, which were not infused with any substrate subtracted from the measured *m*/*z* ratios to correct for natural abundance.

##### Calculation of Enrichments and Fluxes

Whole-body glucose turnover was measured by determining the specific activity of glucose in the steady-state plasma using a scintillation counter. Hepatic glucose production was assumed to represent ∼90% of the measured whole-body glucose turnover based on our previously published data (7). Liver-specific metabolic flux rates were calculated using a combined NMR-LC-MS/MS method. We corrected for the natural abundance of each metabolite included in the flux calculations, measuring all possible enrichments (for instance, m+0, m+1, m+2, m+3, and m+4 for malate) and correcting the measured peak areas to account for the fact that once a carbon is labeled it can no longer contribute to the natural abundance (10). Samples were prepared for NMR by homogenizing 2–3 g of liver in 5 volumes of 7% perchloric acid. The pH of the samples was adjusted to 6.8–7.3 using 30% potassium hydroxide and 7% perchloric acid as necessary, and the samples were centrifuged at 4000 × *g* for 10 min. The supernatant was frozen in liquid N_2_ and lyophilized. ^13^C NMR analysis was performed as described by Befroy *et al.* (6). Total glucose and alanine enrichment was measured by GC/MS and glutamate by LC-MS/MS, with ^13^C NMR used to algebraically divide the total enrichment to determine the enrichment of each carbon of these metabolites.

We calculated the [2-^13^C]malate enrichment by relating the positional enrichments of malate to those measured in glutamate assuming (and validating) full equilibration across fumarase as shown in Equations 1 and 2.

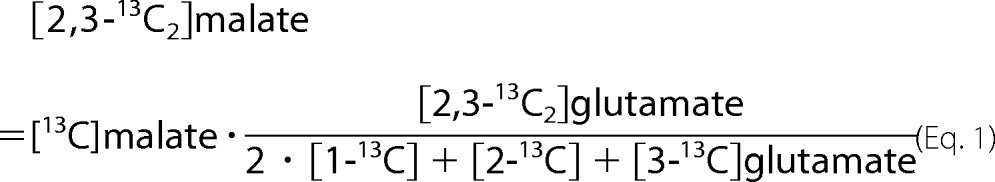


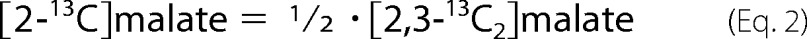


For calculation of the liver-specific metabolic flux ratio *V*_Pyr-Cyc_/*V*_Mito_ = (*V*_PK_ + *V*_ME, out_)/(*V*_PC_ + *V*_ME, in_+ *V*_PDH_), we used our previously published isotopic labeling model (6, 7) extended using a mass isotopomer multiordinate spectral analysis approach to take into account *V*_PK_ and unlabeled mass entry from propionate at the succinyl-CoA step of the TCA cycle (10). Here, *V*_ME, out_ refers to ME flux in the direction of pyruvate synthesis; *V*_ME, in_ refers to the reverse reaction of pyruvate into malate, and *V*_LDH_ refers to pyruvate synthesis via LDH. With these fluxes taken into account, we can describe the steady-state mass balance at pyruvate with Equation 3,

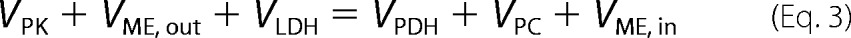
 and isotope balance at [2-^13^C]pyruvate with Equation 4,

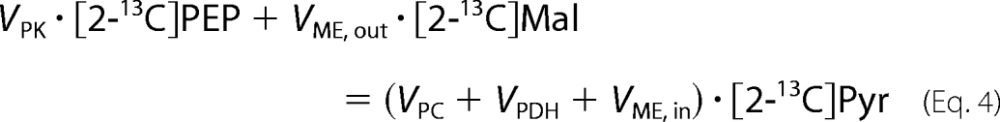


Because the positional enrichments of pyruvate and PEP cannot be measured reliably using our NMR-LC-MS/MS techniques, we use the following label substitutions shown in Equations 5 and 6,

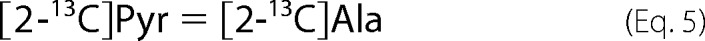


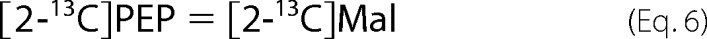


Substituting Equations 5 and 6 into Equation 4 and rearranging, we derive Equation 7,

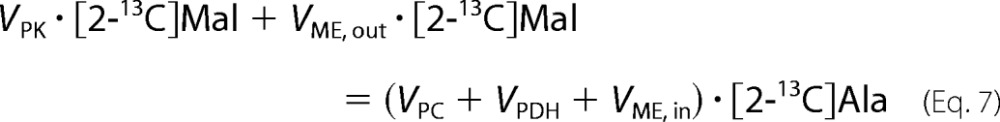


By separating out like terms, we get Equation 8,

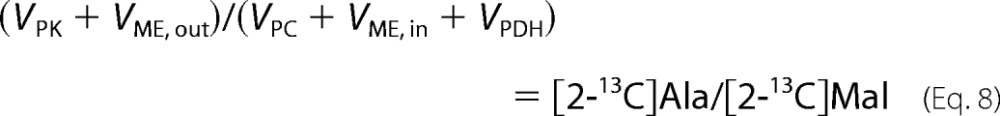
 where *V*_PDH_ denotes flux through pyruvate dehydrogenase; *V*_CS_ denotes flux through citrate synthase; *V*_PK_ denotes flux through pyruvate kinase; *V*_ME, out_ denotes flux through malic enzyme from malate to pyruvate; *V*_ME, in_ denotes flux through malic enzyme from pyruvate to malate; and *V*_PC_ denotes flux through pyruvate carboxylase. Additional fluxes shown in the complete flux diagram in Fig. 1 are *V*_GNG OAA_ gluconeogenesis from oxaloacetate, *V*_GNG_ total gluconeogenesis, and *V*_prop_ the rate of propionate entry into the TCA cycle. *V*_PDH_/*V*_CS_ was measured using the ratio [4-^13^C]glutamate/[3-^13^C]alanine, as described by Alves *et al.* (11).

##### Biochemical Analysis

Plasma glucose concentrations were measured enzymatically using the YSI 2700 Select Biochemistry Analyzer (YSI Inc., Yellow Springs, OH). Plasma lactate was measured by COBAS. Insulin and glucagon concentrations were measured by radioimmunoassay by the Yale Diabetes Research Center RIA Core. Epinephrine was measured by ELISA (MyBiosource). Plasma propionate concentrations were measured by gas chromatography/mass spectrometry (GC/MS) (Hewlett Packard). To 30 μl of plasma, we added an equal volume of [1,2,3-^13^C_3_]sodium propionate (1 m) and acidified with 50 μl of 1 m HCl. After shaking for 20 min, samples were derivatized with 40 μmol of 1,3-dicyclohexylcarlodiimide and 40 μmol of 2,4-difluoroaniline, shaken for 1 h, and dried under N_2_ gas. Samples were then resuspended in ethyl acetate and peak areas (mass to charge ratios: unlabeled propionate, 185; [1,2,3-^13^C_3_]propionate, 188).

##### Tissue Metabolite and Enzyme Analysis

Pyruvate carboxylase activity was measured enzymatically in liver homogenates as Vatner *et al.* (12) have previously described, with propionyl-CoA concentrations in the homogenates matched to those measured *in vivo* by LC-MS/MS.

## Results

### 

#### 

##### Glucagon and Epinephrine Have Differential Effects on V_Pyr-Cyc_/V_Mito_

**FIGURE 2.**
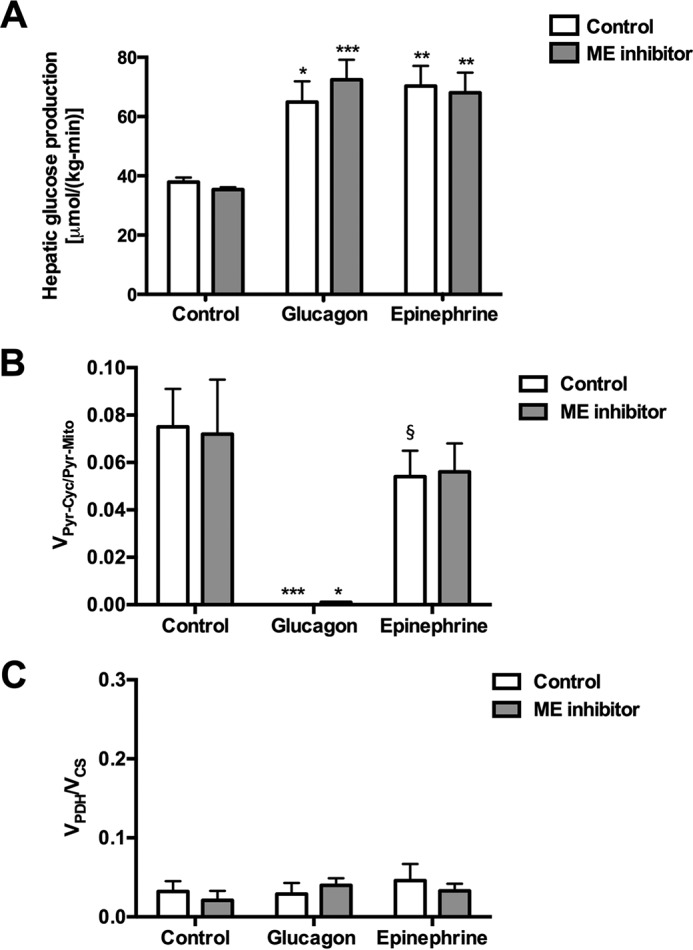
**Physiologic increases in plasma glucagon and epinephrine concentrations both promote increased rates of hepatic glucose production, but only glucagon suppresses *V*_PK + ME_/*V*_PC + PDH_ flux.** *A,* hepatic glucose production. *B,* hepatic *V*_PK + ME_/*V*_PC + PDH_ flux. *C,* hepatic *V*_PDH_/*V*_CS_ flux. In all panels, *, *p* < 0.05; **, *p* < 0.01; ***, *p* < 0.001 *versus* control; §, *p* < 0.05 *versus* glucagon-treated rats. *Symbols* over each *bar* represent comparisons with the analogous group of controls (control or ME inhibitor treated). Data are means ± S.E. of *n* = 6 per group.

A 2-h intra-arterial infusion of either glucagon or epinephrine increased plasma concentrations (within the physiologic range experienced during acute hypoglycemia or stress (13–15)) also raised plasma glucose by 5–8 mm with corresponding increases in plasma insulin (Table 1). Infusion of either hormone increased EGP by ∼70% and was unaffected by malic enzyme inhibition (Fig. 2*A*). Interestingly, glucagon, but not epinephrine, suppressed hepatic *V*_Pyr-Cyc_/*V*_Mito_. Because malic enzyme inhibition did not affect *V*_Pyr-Cyc_/*V*_Mito_, these data suggest that malic enzyme flux is essentially negligible in rats infused with glucagon and epinephrine under fasting conditions (Fig. 2*B*). Neither glucagon nor epinephrine treatment nor malic enzyme inhibition affected the *V*_PDH_/*V*_CS_ flux ratio, which was ∼5% (Fig. 2*C*).

**TABLE 1:** **Glucagon and epinephrine stimulate hepatic glucose production similarly**

Group	Glucose	Insulin	Glucagon	Epinephrine
	*mm*	*pm*	*pm*	*pm*
Control	7.1 ± 0.2	151 ± 28	165 ± 26	1.7 ± 0.4
Control + ME inhibitor	7.1 ± 0.4	178 ± 42	167 ± 20	1.8 ± 1.0
Glucagon	12.9 ± 1.3**	423 ± 86*	934 ± 222**	2.0 ± 1.1
Glucagon + ME inhibitor	14.2 ± 1.1***	529 ± 79**	1012 ± 54****	2.9 ± 0.9
Epinephrine	15.5 ± 1.8***	421 ± 51*	262 ± 57^§§^	8.6 ± 1.1***^§§§^
Epinephrine + ME inhibitor	13.5 ± 1.0***	461 ± 58*	160 ± 14^§§§§^	10.9 ± 1.1^§§§^

*, *p* < 0.05;

**, *p* < 0.01;

***, *p* < 0.001;

****, *p* < 0.0001 *versus* control;

§§, *p* < 0.01;

§§§, *p* < 0.001;

§§§§, *p* < 0.001 *versus* glucagon. Data are means ± S.E. of *n* = 6 per group.

##### Effects of Glucagon and Epinephrine on V_PK_ Are Not Due to Hyperinsulinemia

**FIGURE 3.**
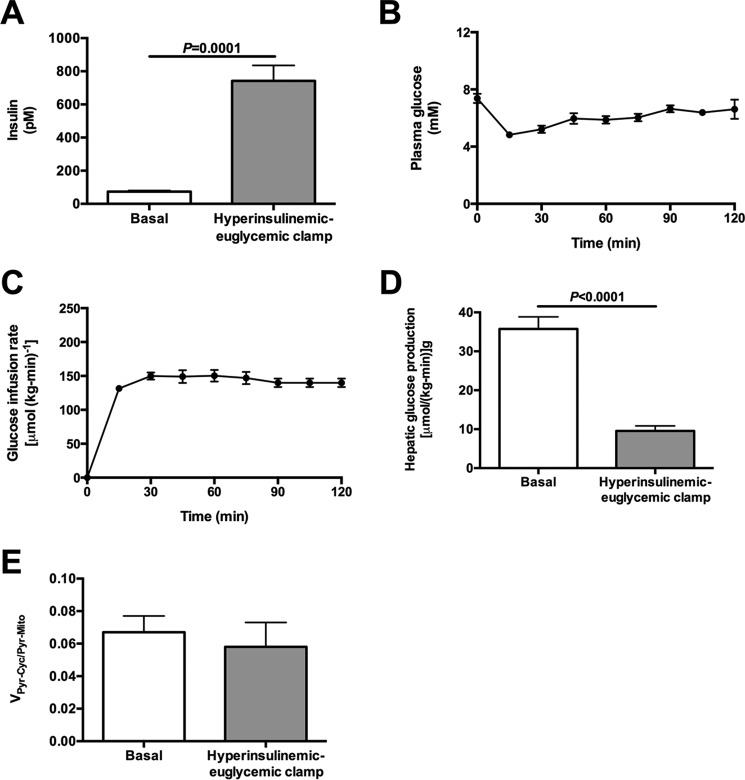
**Hyperinsulinemia cannot explain the changes to fluxes measured in glucagon- and epinephrine-treated rats.** *A,* plasma insulin concentrations after 120 min. *B,* plasma glucose concentrations during the clamp. *C,* glucose infusion rate during the clamp. *D,* hepatic glucose production. *E, V*_PK + ME_/*V*_PC + PDH_ flux, measured as [^13^C_2_]alanine/[^13^C_5_]glucose. Data are mean ± S.E. of *n* = 6 per group.

To determine whether the effects of glucagon or epinephrine on *V*_PK_ were related to the expected hyperinsulinemia following hormone treatment (Table 1), we performed hyperinsulinemic-euglycemic clamps to match plasma insulin concentrations to those measured in the epinephrine- and glucagon-treated rats (Fig. 3, *A–C*). In contrast to glucagon- or epinephrine-treated rats, hyperinsulinemia suppressed hepatic glucose production but did not change *V*_Pyr-Cyc_/*V*_Mito_ (Fig. 3, *D* and *E*).

##### Propionate Stimulates Endogenous Glucose Production and V_Pyr-Cyc_/V_Mito_ in a Dose-dependent Manner

**FIGURE 4.**
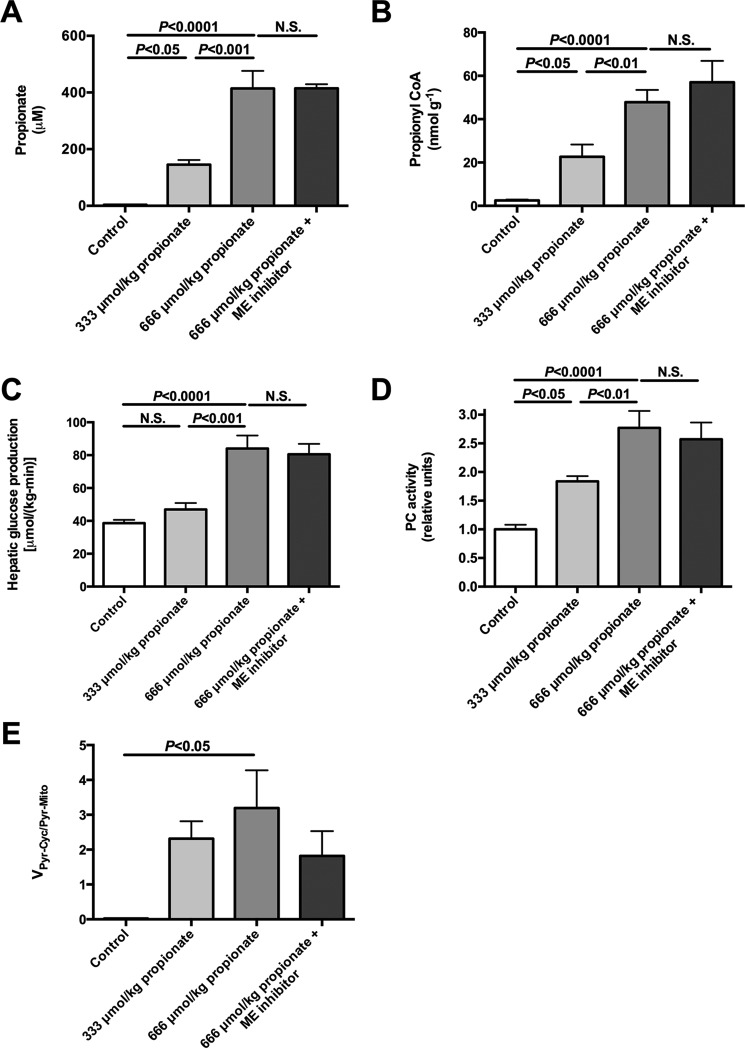
**Continuous intra-arterial propionate infusion (low dose = 333 μmol/kg = 2. 8 μmol/(kg-min) × 120 min; high dose = 667 μmol/kg = 5.6 μmol/[kg-min × 120 min) markedly increases plasma propionate concentrations, hepatic propionyl-CoA concentrations, hepatic glucose production rates, and stimulates PC activity as well as *V*_PK + ME_/*V*_PC + PDH_ flux in a dose-dependent manner.** *A,* plasma propionate concentrations. *B,* liver propionyl-CoA concentrations. *C,* hepatic glucose production. *D, ex vivo* PC activity. *E, V*_PK + ME_/*V*_PC + PDH_ flux. Data are mean ± S.E. of *n* = 6 per group. *N.S.*, not significant.

**FIGURE 5.**
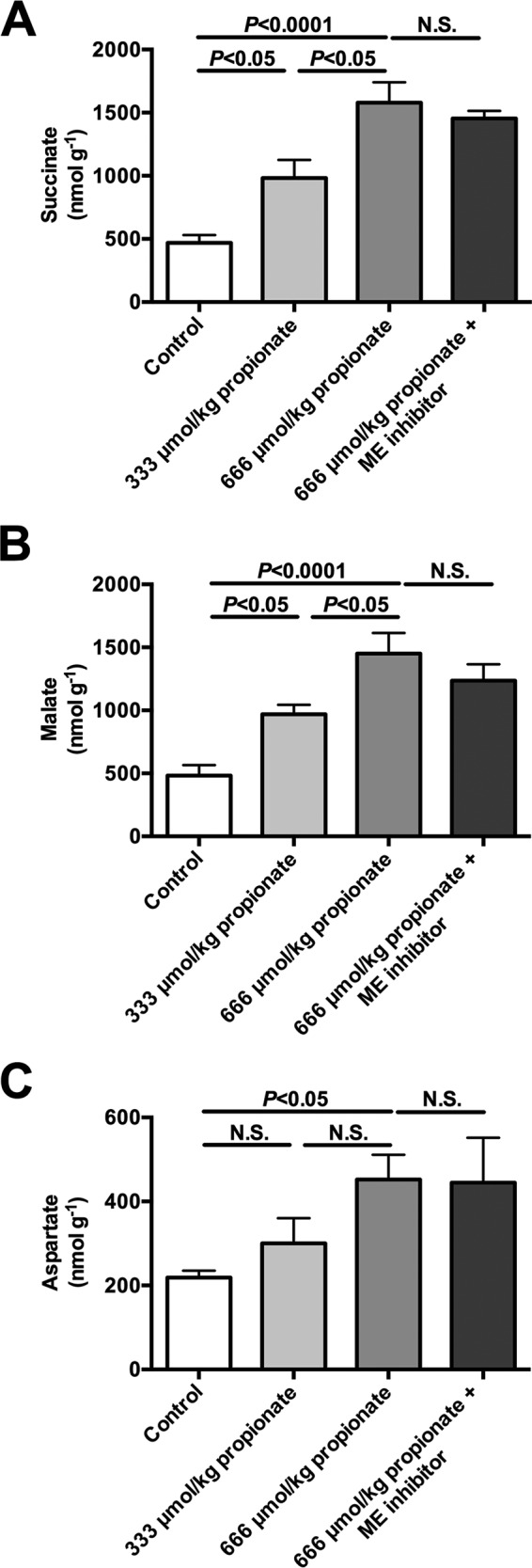
**Continuous intra-arterial propionate infusion (low dose = 333 μmol/kg = 2. 8 μmol/(kg-min) × 120 min; high dose = 667 μmol/kg = 5.6 μmol/[kg-min × 120 min) raises TCA cycle intermediate concentrations in a dose-dependent manner.** *A–C,* liver succinate, malate, and aspartate concentrations. In all panels, data are mean ± S.E. of *n* = 6 per group. *N.S.*, not significant.

**FIGURE 6.**
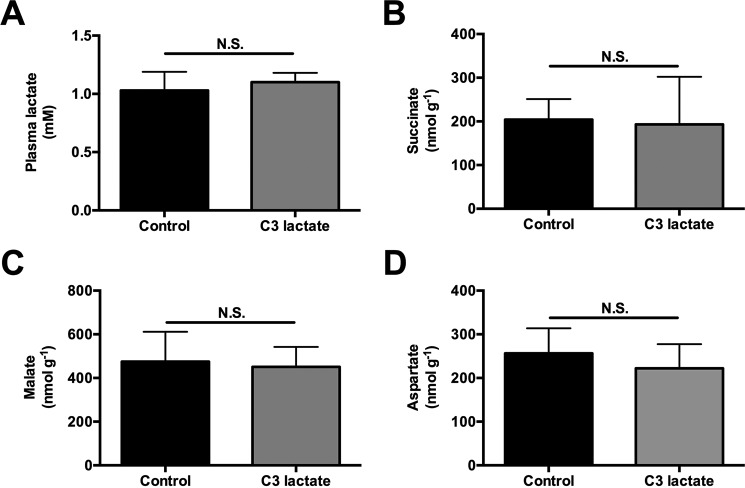
**Infusion of [3-^13^C_3_]lactate (40 μmol/(kg-min) × 120 min) does not change plasma lactate or TCA cycle intermediate concentrations.** *A,* plasma lactate concentrations before (control) or after a 120-min infusion of C3 lactate. *B–D,* liver succinate, malate, and aspartate concentrations. In all panels, data are mean ± S.E. of *n* = 6 per group. *N.S.*, not significant.

To determine whether propionate impacts EGP, glucose turnover was measured during intra-arterial propionate infusion at 2.8 or 5.6 μmol/kg/min (with a total of either 333 or 667 μmol/kg delivered by continuous infusion over 120 min) at rates lower than (2–4, 8) or similar to (5) prior studies. Each dose of propionate raised plasma propionate concentrations 35- and 100-fold, respectively, and hepatic propionyl-CoA concentrations 8- and 18-fold, respectively (Fig. 4, *A* and *B*). These were accompanied by dose-dependent increases in plasma glucose, insulin, and EGP. *Ex vivo* hepatic PC activity was activated (Table 2 and Fig. 4, *C* and *D*) in the presence of these same concentrations of propionyl-CoA similar to previous observations (6, 8). Remarkably, 100–150-fold increases in hepatic *V*_Pyr-Cyc_/*V*_Mito_ flux were also observed in propionate-infused rats (Fig. 4*E*). EGP was not reduced by ME inhibition despite a 40% lowering of *V*_Pyr-Cyc_/*V*_Mito_. Taken together, propionate infusion impacts metabolism and drives flux through PK and/or ME. The increase in EGP and *V*_Pyr-Cyc_/*V*_Mito_ could be explained by a substrate push mechanism, a mechanism supported by the dose-dependent increase in concentrations of TCA cycle intermediates measured in propionate-infused rats (Fig. 5, *A–C*). In contrast, there was no change in plasma lactate or hepatic succinate, malate, or aspartate concentrations following [3-^13^C]lactate infusion at the rates used in our studies to assess*V*_Pyr-Cyc_/*V*_Mito_ (Fig. 6, *A–D*). The ratio of ^13^C enrichments in C2 *versus* C3 glutamate was ∼1.0 (0.95–1.07) in all of the [3-^13^C]lactate-infused groups and confirmed complete equilibration between malate and fumarate in the absence of propionate. However, propionate increased the ratio to 1.22 ± 0.17 suggesting that propionate can also perturb mitochondrial fumarase metabolism out of equilibrium.

**TABLE 2:** **Propionate drives hepatic glucose production in a dose-dependent manner**

Group	Control	333 μmol/kg propionate	667 μmol/kg propionate
Glucose (mm)	7.0 ± 0.3	9.3 ± 0.5	13.4 ± 1.1****^§§^
Insulin (pm)	92 ± 21	338 ± 42*	604 ± 93****^§^

*, *p* < 0.05;

****, *p* < 0.0001 *versus* control;

§, *p* < 0.05;

§§, *p* < 0.01 *versus* low propionate. *n* = 6 per group. Data are means ± S.E.

##### Oral Propionate Increases Liver Propionyl-CoA Concentrations 100-Fold

**FIGURE 7.**
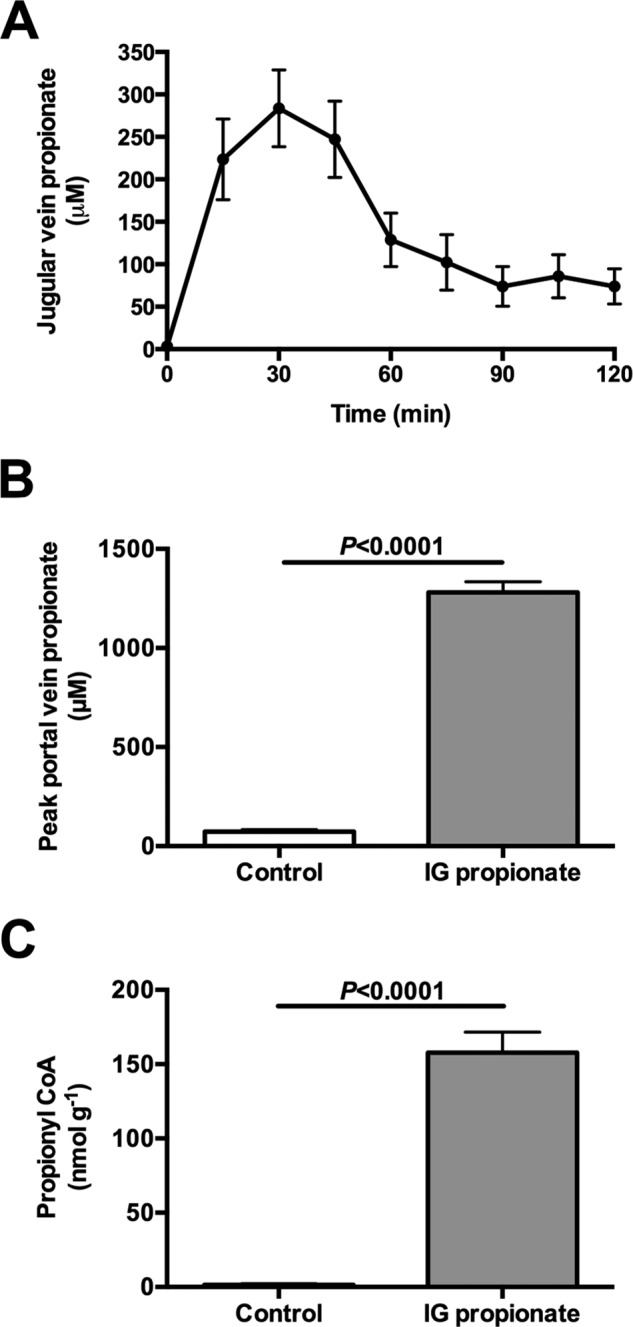
**Oral bolus of propionate (333 μmol/kg) raises portal vein propionate concentrations to 1300 μm and increases hepatic propionyl-CoA concentrations 100-fold.** *A,* jugular vein propionate concentrations. *B,* portal vein propionate concentrations 30 min after an oral bolus. *C,* liver propionyl-CoA concentrations 30 min after an oral bolus. Data are mean ± S.E. of *n* = 6 per group.

Our studies had administered propionate continuously over 2 h, and this protocol will result in lower peak plasma propionate and liver propionyl-CoA concentrations than those measured when propionate is given as a single oral or i.p. bolus (as is typical for human studies of hepatic metabolism) (16). Jugular vein plasma propionate concentrations increased ∼75-fold following administration of an intragastric propionate bolus, with peak portal vein propionate concentrations 4-fold higher than plasma venous concentrations (Fig. 7, *A* and *B*). Furthermore, peak liver propionyl-CoA concentrations increased more than 100-fold following intragastric propionate treatment (Fig. 7*C*).

## Discussion

In principle, the balance between the opposing *V*_PK_ and *V*_PC_ reactions is very important in the regulation of gluconeogenesis. To assess the magnitude of the physiologic contribution of these pathways to metabolic homeostasis, we developed a combined NMR-LC-MS/MS method to directly measure V_Pyr-Cyc_ relative to V_Mito_ *in vivo*. A significant contribution by ME under these experimental conditions was ruled out because *V*_Pyr-Cyc_/*V*_Mito_ did not change in rats treated with a malic enzyme inhibitor. Because hepatic *V*_PDH_ was found to be low relative to *V*_CS_ (<5%) (Fig. 2*C*), *V*_Pyr-Cyc_/*V*_Mito_ can be simplified to *V*_PK_/*V*_PC_. *V*_PK_ rates relative to *V*_PC_ were only ∼6% and suppressed by glucagon, but not epinephrine, consistent with previous data from perfused rat livers (17). Thus under these physiologic conditions, PK only exerted limited control over net gluconeogenic flux. Further studies would be required to demonstrate whether this is also the case in fed animals and under other conditions that might be expected to increase pyruvate kinase flux.

Hepatic *V*_PK_ flux was only ∼6% of *V*_PC_ flux or ∼15% of *V*_CS_ flux based on our previous estimates of *V*_CS_ (Figs. 2*B*, 3*E*, and 4*E*) (6, 7), in stark contrast to the reports of a 2–4-fold higher rate of *V*_PK_ flux relative to *V*_CS_ flux in rats and humans where [1,2,3-^13^C_3_]propionate was used as a tracer (3–5, 16, 18, 19). If true, these high rates of futile cycling would consume most of the ATP produced through oxidative phosphorylation, placing the hepatocyte in a metabolically precarious position. An alternative explanation would be that the infused propionate interferes with metabolism and increases the (real or apparent) rates of futile pyruvate cycling. To test this hypothesis, we infused rats with a total dose of intra-arterial propionate that was lower than (2–4, 8) or similar to (5) the total amount of sodium propionate that was administered as an oral or i.p. bolus in previous studies. Propionate infusion dose-dependently increased plasma propionate concentrations (up to 100-fold), liver propionyl-CoA concentrations (up to 18-fold), *V*_Pyr-Cyc_/*V*_Mito_ (up to 150-fold), and EGP (up to 2-fold) *in vivo* (Fig. 4). Consistent with these results, similar infusion rates of [1,2,3-^13^C_3_]propionate in mice led to substantial glucose enrichment indicating a significant contribution from propionate to gluconeogenic flux (8). However, neither the effects of propionate on the labeling patterns nor the extra mass input by propionate infusion alone could explain the increase in EGP that was measured here; if all of the infused propionate were converted to glucose, this would explain less than 5% of the increase in EGP measured in the propionate-infused rats. These data raised the question of whether propionate itself stimulates EGP beyond its effects to increase this process by mass balance. Interestingly, we found that propionyl-CoA itself was able to double PC activity *in vitro* when applied at the concentrations measured *in vivo* (Fig. 4*D*), consistent with studies by Scrutton *et al.* (18), who found that propionyl-CoA potently stimulated PC activity *in vitro*. Thus, at least in part, PC activation by propionyl-CoA may provide a mechanistic explanation for the propionate-induced increase in EGP measured here. Our data are also consistent with previously observed increases in hepatic citrate synthase flux with increasing doses of propionate (8).

Another potential issue with the use of propionate as a metabolic tracer has to do with the large differential uptake of propionate as it traverses the liver bed, which will also invalidate many of the assumptions involved in the metabolic flux rate calculations. Propionate enters the liver from the portal circulation (from either i.p. or gastrointestinal delivery or arterially (i.v. infusion) and is almost completely cleared from the blood by the time it reaches the hepatic vein (20–22). As such, the periportal hepatocytes take up much more propionate than perivenous hepatocytes. Serious concerns about the use of propionate as a tracer have been raised before because in contrast to other metabolic tracers such as alanine or lactate, propionate is subject to significant hepatic zonation (20–22). The effects of zonation on [1,2,3-^13^C_3_]propionate metabolism by itself could explain much of the wide variation in flux estimates from livers perfused at 0.1 *versus* 0.5 mm where high propionate increased *V*_PEPCK_ flux and *V*_PC_ flux 2–4-fold (4, 19). It should also be noted that the impact of zonation on estimates of hepatic metabolic fluxes will become even greater with lower doses of [1,2,3-^13^C_3_]propionate.

In addition to these concerns, we also found that infusion of propionate induced dose-dependent 2–3-fold increases in the concentration of hepatic TCA intermediates (malate and succinate) and aspartate, which will impact hepatic mitochondrial metabolism. The propionate-induced increase in hepatic malate concentrations likely shifts the equilibrium balance across ME that could increase exchange or net flux into pyruvate; indeed, propionate has previously been shown to increase this flux (23). Similar data have been obtained in heart, where propionate activates PDH flux, consistent with a large increase in futile cycling, and it increases TCA cycle intermediate concentrations (24).

Concerns have also been raised regarding lactate as a tracer (4, 25, 26) suggesting, among other things, that disequilibrium across fumarase may underestimate pyruvate cycling rates in the lactate method used here. However, direct ^13^C NMR measurements of the [2-^13^C]glutamate to [3-^13^C]glutamate ratio were ∼1.0 (0.95–1.07) in control and glucagon- and epinephrine-treated rats. In contrast, propionate increased this ratio (1.22 ± 0.17). These data directly refute the claim that [3-^13^C]lactate promotes disequilibrium across fumarase except in the case where it is combined with propionate. Furthermore, in contrast to propionate, lactate infusion does not raise plasma lactate concentrations (Fig. 6*A*), and doubling the rate of [3-^13^C]lactate infusion does not significantly impact EGP, *V*_PC_/*V*_CS_, or *V*_PDH_/*V*_CS_ (6).

Several studies utilizing [1,2,3-^13^C_3_]propionate as a metabolic tracer administered the propionate orally (27, 28) or by i.p. boluses (2, 3). Unfortunately, these studies did not measure plasma concentrations or plasma ^13^C enrichments of propionate following [1,2,3-^13^C_3_]propionate administration, so it is unclear whether they achieved steady plasma propionate concentrations and/or steady-state plasma propionate ^13^C enrichments in their studies, which is a critical assumption in their model. In our studies, we infused similar total doses of propionate to those used in these previous studies. As our studies employed intra-arterial infusions at a constant rate, they would not be expected to raise portal propionate concentrations to the same extent that an oral or i.p. propionate injection would; thus, if anything, our results would be expected to underestimate both the exposure and impact of this same dose of propionate on liver metabolism compared with these previous studies (2, 3, 27, 28). Consistent with this hypothesis, we administered the same dose of propionate as an oral bolus and observed peak portal vein concentrations of propionate of ∼1.2 mm that were 3–4-fold higher than jugular vein propionate concentrations and peak propionyl-CoA concentrations that were also 3–4-fold higher than when the same dose of propionate was administered by a continuous infusion.

Taken together, our data demonstrate that propionate significantly alters hepatic flux rates, including gluconeogenesis and pyruvate recycling via pyruvate kinase, when administered at levels used in previous [1,2,3-^13^C_3_]propionate tracer studies. In contrast to previous studies in which [1,2,3-^13^C_3_]propionate was used as a tracer, we found that rates of pyruvate recycling via PK and ME were minimal under physiologic fasting conditions and with infusions of epinephrine and glucagon. The large impact of propionate on these fluxes and mitochondrial intermediate concentrations stands in direct contrast to the minimal impact observed with the use of lactate as a tracer of hepatic glucose and mitochondrial metabolism (Fig. 5, *A–C*) (6). With the possible exception of conditions in which propionate concentrations are supraphysiologic (for instance, propionic acidemia) or in studies of ruminants, propionate administered at these previously described doses will perturb metabolism to an extent that renders the results of metabolic studies using [1,2,3-^13^C_3_]propionate as a metabolic tracer uninterpretable.

In summary, we demonstrate that propionate, at previously described doses, is an unsuitable tracer to assess hepatic metabolism due to its profound modulating effects on hepatic TCA cycle metabolite concentrations as well as on gluconeogenesis and pyruvate kinase flux rates in awake rodents *in vivo*. It is anticipated these same observations will apply to studies in human subjects.

## Author Contributions

Experiments in this report were performed by R. J. P., C. B. B., X.-M. Z., D. Z., T. C. A., and G. W. C. K. F. P. participated in method development and data analysis. D. L. R., R. G. K., and G. I. S. derived the equations used for flux analysis. Studies were designed, data were analyzed, and the manuscript was written by R. J. P., R. G. K., and G. I. S. with input from all authors.

## Footnotes

3 The abbreviations used are:

PK: pyruvate kinase
CS: citrate synthase
EGP: endogenous glucose production
ME: malic enzyme
PC: pyruvate carboxylase
PDH: pyruvate dehydrogenase
PEP: phosphoenolpyruvate
TCA: tricarboxylic acid.
